# Effect of Nb-Ti Microalloyed Steel Precipitation Behavior on Hot Rolling Strip Shape and FEM Simulation

**DOI:** 10.3390/ma17030651

**Published:** 2024-01-29

**Authors:** Kaisheng Li, Jian Shao, Chihuan Yao, Pan Jia, Shuhao Xie, Desheng Chen, Min Xiao

**Affiliations:** 1 National Engineering Research Centre for Advanced Rolling Technology, University of Science and Technology Beijing, Beijing 100083, China; m202111241@xs.ustb.edu.cn (K.L.); yaochihuan@outlook.com (C.Y.); panda_jasper@163.com (P.J.); bk_xieshuhao@163.com (S.X.); cds18131055619@163.com (D.C.); 2 Digital Intelligence Department, Xinyu Iron and Steel Co., Ltd., Xinyu 338001, China; xm@xinsteel.com.cn

**Keywords:** microalloyed steel, finish rolling, precipitation, strip shape, control optimization

## Abstract

Strip shape control is a hotspot and challenge in strip rolling, where the development trend of rolling technology is towards high strength, high toughness, and a large width-to-thickness ratio. The influence of material microstructure evolution on strip shape control is being increasingly emphasized. In this paper, a Nb-Ti microalloyed steel is taken as the research object. Thermodynamic and kinetic models focusing on the precipitation of the austenite phase are established to quantify the precipitation process. A coupled model of rolls and strips is built using ABAQUS 2022 software, where the precipitation strengthening model and high-temperature constitutive model are embedded into the finite element model (FEM) through subroutines. A two-dimensional alternating differential model is employed to acquire real-time temperature differences in the width direction of the strip. The effects of precipitation inclusion and exclusion on the strip crown under different operating conditions are compared and analyzed. The results indicate that as the temperature decreases, the strengthening effect increases, reaching around 40 MPa at temperatures above 1000 °C and 96.6 MPa at 800 °C. Furthermore, the inclusion of crown in the precipitation consideration is more sensitive to overall temperature changes, but as the strip width decreases, the sensitivity of crown to temperature decreases. The research findings of this paper provide guidance for improving strip shape control and reducing abnormalities during the rolling process.

## 1. Introduction

With the development of the economy and society, improving the strength of steel components and reducing the weight of steel products have become key indicators for the iron and steel industry’s development. Consequently, the industry has been focusing on the development of new high-strength steel [1,2,3]. Microalloyed steel, characterized by high yield strength, high toughness, and high durability, is achieved by incorporating small amounts of alloying elements (such as Nb, Ti, and V) into the chemical composition of ordinary mild steel and ordinary high-strength low-alloy steel matrices, thereby enhancing the mechanical and corrosion resistance properties of the steel [4]. Due to the exceptional performance of microalloyed steel, it has been widely utilized in everyday life.

**Figure 1:**
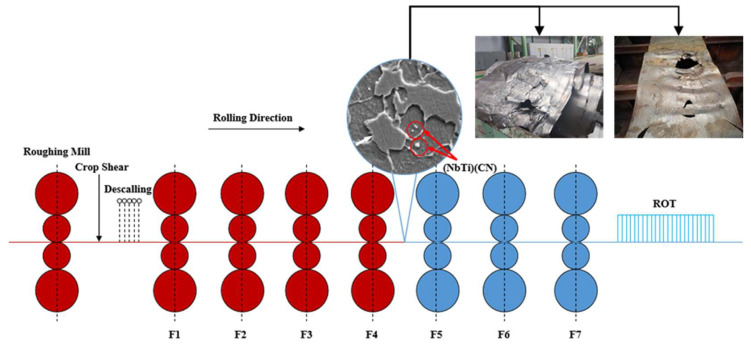
The precipitation behavior during the precision rolling of microalloyed steel and its impact on the strip shape.

However, when producing thin-specification microalloyed strip steel with a strength level of over 700 MPa, temporary changes in working conditions such as rushed production schedules, insufficient combustion in heating furnaces, and abnormalities in the rolling process can lead to a decrease in the steel’s temperature control capability. This results in significant longitudinal and transverse temperature differentials, which can then cause accidents such as steel stack-ups and tail throwing between the mill stands [5], as shown in Figure 1. However, due to the unique characteristics of the material composition that affect product performance, the final temperature overlaps with the precipitation temperature of microalloyed steel. Therefore, the precipitation behavior of the second-phase particles will occur during the finishing rolling process. Additionally, the significant temperature difference in the transverse direction of the strip steel during widening, along with the high sensitivity of precipitation to temperature, leads to a nonuniform size and volume fraction of the precipitates. The transverse variation in the precipitates results in an uneven distribution of material deformation resistance, reducing the prediction accuracy of the traditional austenite rolling model, which neglects the influence of precipitation. This nonuniformity in the hot rolling process of microalloyed steel can cause excessive thickness or abnormal fluctuations, severely impacting product quality and stability. The purpose of this paper is to investigate the influence of the precipitation behavior of microalloyed high-strength steel on the hot rolling strip shape.

The issue of strip shape in hot rolling is of utmost importance in terms of quality and is also one of the most important indicators in the production process. Many scholars have approached this issue through the study of rolling processes, utilizing machine learning and deep learning methods [6,7,8,9] to predict the influencing factors on strip shape. However, such methods overlook the impact of material properties on strip shape, which can lead to inaccurate predictions when faced with unique circumstances. In addition, FEM is also a commonly used approach that considers material properties and can be employed to simulate actual rolling conditions during the production process. For example, Rumualdo Servin et al. [10] utilized finite element model (FEM) analysis to explore the influence of crown on the stress distribution and elastic deformation during rolling, while Zhao J. W. et al. [11] quantitatively studied the impact of metal lateral flow and stress relaxation on the strip shape in the context of thermal rolling. However, the causes of strip shape issues are highly complex, and different strip shape problems can be caused by different factors. Therefore, the combination of material properties and strip shape issues, along with the analysis and treatment tailored to specific conditions [12], serves as the foundation for further resolving strip shape problems with the assistance of the convenience offered by FEM. For silicon steel, due to its high phase transformation temperature and fast phase transformation rate, Li W G et al. [13] proposed a rolling force model that considers phase transformation based on the deformation resistance change in the dual-phase region during rolling. Building upon this, Liu C et al. [14] further took into account the influence of uneven temperature distribution on phase transformation and studied the impact of non-uniform internal stress and microstructural differences caused by phase transformation on strip shape in non-oriented silicon steel. For microalloyed high-strength steel, precipitation strengthening plays an extremely vital role in influencing the shape of the strips during the hot rolling process. However, there is currently a lack of research on the effect of precipitation strengthening on the shape of hot rolling strips.

The primary concern in the process of precision rolling involves the phenomenon of strain-induced precipitation, and many scholars have studied it [15,16,17]. Dutta and Sellars [18] were the first to propose a precipitation model for Nb microalloyed steels, incorporating the influences of material deformation and recrystallization factors. Building upon this foundation, Liu W. J. et al. [19] introduced a new kinetic model for strain-induced precipitation by considering the effects of non-equilibrium segregation and dislocations. Subsequently, Dutta B. [20] further refined the influence of dislocations based on previous research, establishing a generalized precipitation kinetics model. However, there are many factors influencing strain-induced precipitation that warrant further investigation. Benoit, M. J. et al. [21] studied the effects of additive manufacturing processes and isothermal aging on the microstructure and properties of 13-8 Mo precipitation hardening martensitic stainless steel. Wang L. C. et al. [22] utilized thermodynamic calculation software to explore the influence of microalloying elements on the precipitation phases in austenitic stainless steels. As research on microalloyed steels continues to mature, steel compositions with different alloying element contents have gradually been introduced into production, drawing significant attention to associated production issues. Among them, Varanasi, R. S. et al. [23] investigated the influence of 0.06 wt.% Nb micro-alloying in medium manganese steel of composition 10Mn-0.05C-1.5Al (wt.%). Qu J. B. et al. [24] developed mathematical models that describe various metallurgical phenomena during controlled rolling and controlled cooling processes of HSLA (high-strength low-alloy) steels, incorporating kinetic models into mechanical performance prediction models. Patra, P. et al. [25] studied the effect of coiling temperature on the microstructure and mechanical properties of hot-rolled Ti-Nb microalloyed ultra-high-strength steel. However, in the current research on precipitation strengthening during the production process of microalloyed steels, the focus of researchers has primarily been on the understanding of the strengthening mechanisms and the investigation of mechanical properties. However, there still remains a knowledge gap when it comes to the evolution of precipitation strengthening during the precision rolling process and its impact on the shape of hot rolling strips.

This paper focuses on two kinds of Nb-Ti microalloyed steels and establishes a precipitation thermodynamic model and a kinetics model with austenite precipitation as the research object to quantify the precipitation process. The model is validated through simulations and experiments. Then, a roll system-rolling piece coupling model is built using ABAQUS 2022 software, and the experimentally obtained precipitation strengthening model and high-temperature constitutive model are incorporated into the FEM model using subroutines. The influences of considering and not considering precipitation on the strip’s crown under different conditions are compared and analyzed. The study reveals the impact of precipitation strengthening on the hot rolling deformation behavior, laying the theoretical foundation for further development of an accurate predictive model for hot rolling microalloyed steels.

## 2. Materials and Methods

This section describes the experimental materials and methods involved in this paper. The Gleeble 3500 (Dynamic System Inc., New York, NY, USA)was used to obtain stress–strain curves under different working conditions through high-temperature compression tests. The kinetics of the precipitation model can be verified by stress relaxation experiments. The samples obtained from the experiments can be observed for microstructure using a scanning electron microscope (SEM), and the chemical composition of the precipitates can be analyzed using an X-ray energy dispersive spectrometer. The morphology and size of the precipitated phases at different high temperatures are recorded. Combined with observations and theoretical models from previous studies, the precipitation-strengthening effect is quantified and compared with the high-temperature constitutive model to form a control group in preparation for the next step of simulation and modeling.

### 2.1. Experimental Materials

According to the production situation of a certain company, this paper selects two types of experimental steels (1# and 2#), both of which are Nb-Ti microalloy high-strength steels with a strength of over 700 MPa. The chemical composition is shown in Table 1.

### 2.2. High-Temperature Compression Experiment

In order to simulate the hot rolling process, by conducting a high-temperature compression experiment, stress–strain curves of samples under different conditions can be obtained. This enables the analysis of the effect of various factors on the deformation resistance of the sample at high temperatures, as well as the determination of material parameters for high-temperature constitutive models. These parameters can be used to study the influence of precipitate behavior on the mechanical properties during the fine-rolling process.

**Figure 2:**
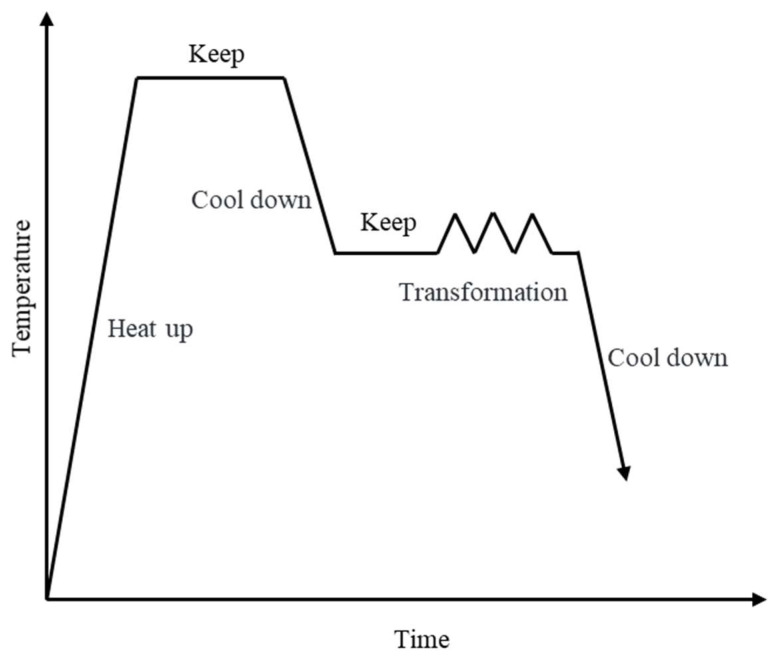
Schematic diagram of high-temperature compression experiment.

As shown in Figure 2, to begin with, the sample is heated to 1200 °C at a rate of 10 °C/s and held for 5 min to ensure complete austenitization. It is then cooled to the desired temperature (800 °C, 850 °C, 900 °C, 950 °C, 1000 °C, or 1050 °C) at a rate of 5 °C/s and held for 30 s to eliminate temperature gradients. Subsequently, the compressed deformation is performed at a set strain rate and strain rate of 1 s^−1^ and 60% strain, respectively. After compression, the sample is water-cooled to room temperature.

**Figure 3:**
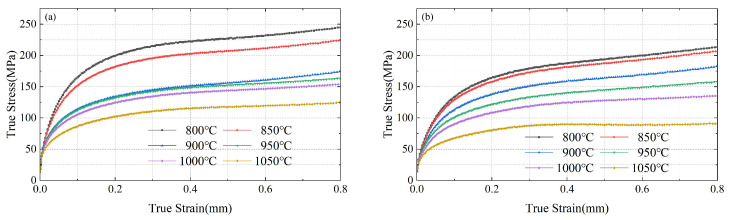
Stress–strain curves of samples 1# (**a**) and 2# (**b**) at different temperatures.

As shown in Figure 3, it can be observed that due to limitations of lubrication conditions, the sample exhibits a bulging phenomenon, leading to an upward trend of the stress–strain curve at higher strains. This increases the required pressure during compression. Therefore, when analyzing the true stress–strain curve, experimental data below a strain of 0.6 are considered. According to Figure 3, it can be seen that, due to the significant difference in Nb and Ti element content, when the temperature is relatively low and the true strain is the same, the true stress of sample 1# (a) can be approximately 20% greater than that of sample 2# (b).

### 2.3. Stress Relaxation Experiment

**Figure 4:**
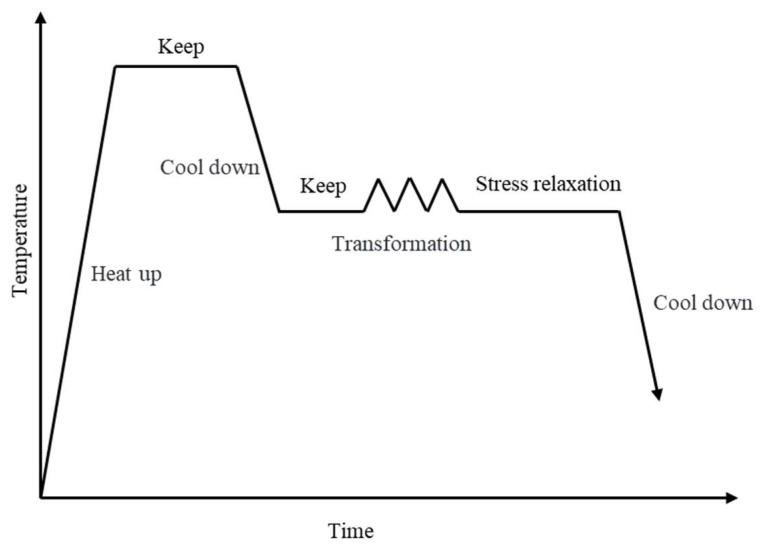
Schematic diagram of stress relaxation experiment.

As shown in Figure 4, the stress relaxation curves of samples 1# and 2# after 20% deformation at 800 °C~1050 °C and isothermal relaxation for 2000 s are obtained. The specific operation is as follows: first, the sample is heated to 1200 °C at a heating rate of 10 °C/s and then held for 5 min to fully austenitize the sample. Then, the sample is cooled to the deformation temperature at a cooling rate of 5 °C/s, and held for 30 s to balance the temperature difference. Subsequently, the sample is compressed with a strain rate of 1 s^−1^ and a deformation amount of 20%, consistent with the high-temperature compression experiment, followed by isothermal stress relaxation for 2000 s. After the experiment, the sample is immediately quenched in water, and the experimental results will be presented in Section 3.2.

### 2.4. Microscopic Observation and Analysis

**Figure 5:**
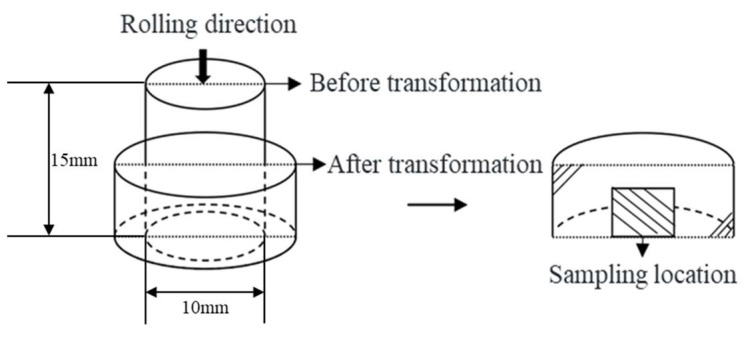
Sample shape and sampling position.

The center of the sample after stress relaxation is the microscopic observation area of the microstructure. Before observing the sample, sampling, embedding, grinding, polishing, and etching operations are required. First, sampling is conducted, and the sample deformation before and after and the sampling schematic diagram are shown in Figure 5. The sample is embedded in plastic using an embedding machine, and then grinding and polishing are performed. Finally, the surface is corroded with a reagent of hydrochloric acid (10%) and ethanol (90%), dried with a blow dryer, and then microscopic observation can be carried out.

## 3. Precipitation Theory Model

The types and contents of microalloying elements in microalloyed steels result in variations in the precipitation process and types of precipitates. Precipitation during the finish rolling process directly affects the size and volume fraction of precipitates, thereby influencing the distribution of the microstructure and mechanical properties of strip steels and ultimately influencing strip shape. In this section, a thermodynamic model for precipitation, a kinetic model for precipitation, and a high-temperature constitutive equation based on the Arrhenius equation have been established to quantify the precipitation process. Combined with the construction of the temperature field, the deformation resistance of microalloyed steels at different temperatures and locations can be obtained, laying the foundation for subsequent simulations using the ABAQUS 2022 software.

### 3.1. Precipitation Thermodynamic Model

In Nb-Ti microalloyed high-strength steel, the typical precipitates observed include TiC, Ti(CN), NbC, Nb(CN), and (NbTi)(CN). These precipitates form due to the structural similarities between the carbides and nitrides of the Nb and Ti elements. They readily dissolve into each other, leading to the formation of composite compounds. When assuming an ideal solution within the precipitates, the chemical composition of each component in the precipitates aligns with the ideal chemical ratio [12]. This assumption allows for a simplified description of the chemical composition and behavior of the precipitates in the Nb-Ti microalloyed steel.

Based on the reasonable assumptions mentioned above, the precipitates can be represented as (Nb*_x_*Ti*_y_*)(C*_a_*N*_b_*), where the variables *x*, *y*, *a*, and *b* are the molar fractions of Nb, Ti, C, and N elements, respectively. The constraints for these variables are 0 ≤ *x* ≤ 1, 0 ≤ *y* ≤ 1, 0 ≤ *a* ≤ 1, 0 ≤ *b* ≤ 1, *x + y* = 1, and *a* + *b* = 1. Since NbC, NbN, TiC, and TiN share the same crystal structure, the molar ratio of the compound (Nb*_x_*Ti*_y_*)(C*_a_*N*_b_*) can be considered as a combination of Nb and Ti carbides and nitrides: *xa* mol of NbC, *xb* mol of NbN, *ya* mol of TiC, and *yb* mol of TiN. The thermodynamic equilibrium equation for the carbide/nitride (Nb*_x_*Ti*_y_*)(C*_a_*N*_b_*) can be expressed by the following set of Equation (1) [26].

(1) $$\left\{\begin{array}{l} a\ln\frac{xaK_{NbC}}{x_{Nb}x_{C}}+b\ln\frac{xbK_{NbN}}{x_{Nb}x_{N}}+ab\frac{L_{CN}}{RT}=0\\ a\ln\frac{yaK_{TiC}}{x_{Ti}x_{C}}+b\ln\frac{ybK_{TiN}}{x_{Ti}x_{N}}+ab\frac{L_{CN}}{RT}=0\\ ya\ln\frac{xx_{Ti}K_{NbC}}{yx_{Nb}K_{TiC}}+b\ln\frac{xbK_{NbN}}{x_{Nb}x_{N}}+a^{2}b\frac{L_{CN}}{RT}=0\\ x_{Nb}^{0}=(\frac{x}{2})f_{\left(Nb_{x}Ti_{y})(C_{a}N_{b}\right)}+(1-f_{\left(Nb_{x}Ti_{y})(C_{a}N_{b}\right)})x_{Nb}\\ x_{Ti}^{0}=(\frac{1-x}{2})f_{\left(Nb_{x}Ti_{y})(C_{a}N_{b}\right)}+(1-f_{\left(Nb_{x}Ti_{y})(C_{a}N_{b}\right)})x_{Ti}\\ x_{C}^{0}=(\frac{a}{2})f_{\left(Nb_{x}Ti_{y})(C_{a}N_{b}\right)}+(1-f_{\left(Nb_{x}Ti_{y})(C_{a}N_{b}\right)})x_{C}\\ x_{N}^{0}=(\frac{1-a}{2})f_{\left(Nb_{x}Ti_{y})(C_{a}N_{b}\right)}+(1-f_{\left(Nb_{x}Ti_{y})(C_{a}N_{b}\right)})x_{N} \end{array}\right.$$

where *L_CN_* is the interaction parameter, *K_AB_* is the solubility product of precipitate *AB,* for example, *K_NbC_* represents solubility product of the precipitate *NbC*, *x_A_* is the molar fraction of solute component *A,* for example, *x_Nb_* represents molar fraction of the precipitate solute component *Nb*, $x_{A}^{0}$ is the initial molar fraction of element *A*, for example, $x_{Nb}^{0}$ represents initial molar fraction of element *Nb,* and *f* is the molar fraction of the precipitate phase, respectively. The set of equations consists of 7 unknowns and 7 equations. Before solving the system of equations, certain input data for the equations need to be determined, including the initial mole fractions $x_{Nb}^{0}$ , $x_{Ti}^{0}$ , $x_{C}^{0}$ , and $x_{N}^{0}$ , as well as the temperature *T* and solid solubility product *K_MX_*.

The chemical driving force for the precipitation of the phase with a given solubility product and nucleation rate per unit volume can be determined using Equation (2), where *A* and *B* are constants; [*M*] and [*X*] represent the weight fractions of the dissolved components; *X_i_* is the equilibrium concentration of element *i* at temperature *T*; and *X_i_*_0_ is the concentration of element *i* dissolved during solid solution processing; *V_P_* is the molar volume of the precipitated compound.

(2) $$\left\{\begin{array}{l} \log_{10}^{K_{MX}}=\log_{10}^{\left[M][X\right]}=B-A/T\\ \Delta G_{V}=RT[\ln(X_{Nb}X_{Ti}X_{C}X_{N})-\ln(X_{Nb0}X_{Ti0}X_{C0}X_{N0})]/V_{P}\\ V_{P}=NbC\%V_{NbC}+TiC\%V_{TiC}+NbN\%V_{NbN}+TiN\%V_{TiN} \end{array}\right.$$

Thermo-Calc 2015b software and stress relaxation experiments were employed to verify the thermodynamic and kinetic models of precipitation. The study utilized the TCFE7: Steels/Fe-Alloys v7.0 database in Thermo-Calc 2015b software [27] for thermodynamic calculations of Nb-Ti microalloyed high-strength steel. The accuracy of the theoretical model was validated by calculating the component composition, molar fractions, and other parameters of the precipitated phase. The influence of different conditions on the precipitated phase was analyzed.

**Figure 6:**
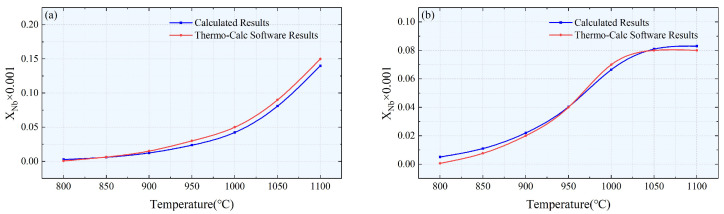
Comparison of the Nb element molar fractions dissolved in the austenite matrix for samples #1 (**a**) and #2 (**b**).

**Figure 7:**
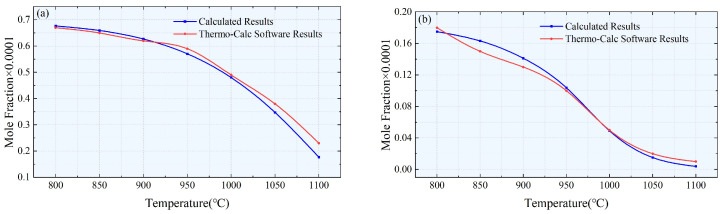
Comparison of equilibrium molar fractions of precipitated phases for samples #1 (**a**) and #2 (**b**).

The results in Figure 6 and Figure 7 compare the Nb element molar fraction in austenite obtained from the theoretical model and Thermo-Calc 2015b software with the equilibrium molar fraction of precipitates at different temperatures for specimens #1 and #2. The small deviations may be attributed to the initial concentration calculations. Additionally, it is important to note that the calculation model only considered four elements: Nb, Ti, C, and N, while neglecting the influence of other elements. Despite these limitations, overall, our research results confirm the accuracy and reliability of the proposed calculation model.

### 3.2. Precipitation Kinetics Model

The precipitation kinetic model is a mathematical model that describes the evolution of the precipitation formation process in materials over time. Based on thermodynamic calculations, the changing patterns of the volume fraction of precipitates with respect to time at different temperatures are obtained, thus obtaining the kinetics curve of precipitation [26].

The critical free energy is an important factor influencing the precipitation of the precipitate phase. It can be calculated using Gibbs’ treatment. Under homogeneous nucleation conditions, considering the chemical free energy change Δ*G_chem_* during nucleation, the interfacial energy change Δ*G_int_* associated with precipitate formation, and the strain energy change Δ*G_dis_* caused by nucleation, the system’s free energy change resulting from the formation of a nucleus with a radius *r* can be described as Equation (3).

(3) $$\left\{\begin{array}{l} \Delta G=\Delta G_{chem}+\Delta G_{dis}+\Delta G_{\mathrm{int}}\\ \Delta G_{chem}=\frac{4}{3}\pi r^{3}\Delta G_{V}\\ \Delta G_{dis}=-0.4\mu b^{2}r\\ \Delta G_{\mathrm{int}}=4\pi r^{2}\sigma \end{array}\right.$$

where *μ* is the shear modulus of the carbonitride and *b* is the Burger vector of the dislocation, respectively. Taking the derivative of Δ*G* with respect to *r* and setting it equal to 0 yields the values of the critical nucleation free energy and critical radius for dislocation nucleation.

When considering dislocation nucleation, assuming a constant nucleation rate during the precipitation process, the steady-state nucleation rate can be calculated using the following Equation (4) [28].

(4) $$\left\{\begin{array}{l} I_{pre}=\frac{D_{M}x_{M}}{a^{3}}\rho \exp(-\frac{\Delta G^{*}}{kT})\\ D_{M}=xD_{Nb}+yD_{Ti}\\ x_{M}=xx_{Nb}+yx_{Ti}\\ \Delta G^{*}=16\pi \sigma^{3}/3\Delta G_{V}^{2} \end{array}\right.$$

where *a* is the lattice constant of austenite, which is 3.646 × 10^−10^ m, *k* is the Boltzmann constant, and *ρ* is the dislocation density, which is 1.38 × 10^−23^ J/K, respectively.

The temporal evolution of the dimensions of carbon nitride particles, characterized by the radius *r* and the volume fraction *Y*, can be represented as shown in Equation (5).

(5) $$\left\{\begin{array}{l} r(t)=\sqrt{2\frac{C_{M}^{0}-C_{M}^{\gamma }}{C_{M}^{p}-C_{M}^{\gamma }}D_{M}t}\\ Y=1-\exp\{-\frac{16}{15}\pi {D_{M}}^{3/2}\alpha_{pre}I_{pre}t^{5/2}\} \end{array}\right.$$

where $C_{M}^{0}$ , $C_{M}^{\gamma }$ and $C_{M}^{p}$ are the equilibrium volume concentrations of solute elements in the matrix far away from the precipitate, solute elements at the austenite side of the precipitate, and solute elements at the precipitate side, *α_pre_* is the growth rate of the precipitate phase, respectively. By relating the time and volume fraction of the precipitate, the corresponding precipitation-time–temperature (PTT) curve can be plotted.

**Figure 8:**
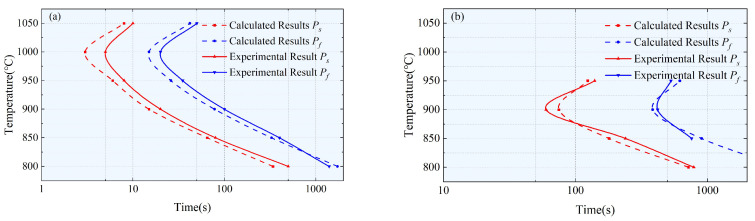
PTT curves of samples #1 (**a**) and #2 (**b**).

Figure 8 shows that the theoretical results and experimental results are in good agreement with *P_s_* and *P_f_* representing the start time and finish time of precipitation, respectively. Both Samples #1 and #2 exhibit a typical “C”-shaped PTT curve, with a nose temperature of 1000 °C for Sample #1 and 900 °C for Sample #2. The formation of precipitated phases is a spontaneous process, and the fastest precipitation temperature is determined by both thermodynamics and kinetics. When the content of Nb and Ti increases, the lattice constant of the alloy also increases, leading to an increased degree of lattice mismatch between the precipitated phases and the matrix. This increases the difficulty in kinetics, requiring higher degrees of superheating and the fastest precipitation temperature. In the stress relaxation diagram of Sample #2 at 800 °C, the corresponding precipitation end time *P_f_* was not observed. This is because the set relaxation time of 2000 s for Sample #2 at 800 °C is too short. In the calculation model, it can be determined that the corresponding end time is approximately 3600 s.

### 3.3. Precipitation Strengthening and High-Temperature Constitutive Models

The precipitation strengthening effect primarily depends on the size, distribution, and volume fraction of the particles. The precipitation strengthening effect calculated using the Orowan mechanism can be described as Equation (6) [29].

(6) $$\sigma_{Orowan}=\frac{\sqrt{6}\mu b}{1.18\pi^{3/2}k_{p}d_{p}}{f_{p}}^{1/2}\ln(\frac{\pi k_{d}d_{p}}{4b})$$

where *f_p_* is the volume fraction of the precipitate phase, *d_p_* is the average diameter of the precipitate phase, *k_p_* is the proportionality constant, which is 0.8, *k_d_* is the correction parameter for the size of the precipitate phase, which is 1.1, *μ* is the shear coefficient, which is 80,260 MPa, and *b* is the Burgers vector, which is 0.248 nm, respectively.

Using the Arrhenius equation to establish a high-temperature constitutive model, the Arrhenius equation has three forms: power-exponential, exponential, and hyperbolic-sine, corresponding to low-stress conditions, high-stress conditions, and all stress conditions, respectively. The effects of deformation temperature and deformation rate on deformation can be comprehensively represented by the parameter *Z*. As shown in Equation (7) [30].

(7) $$\left\{\begin{array}{l} \ln\dot{\varepsilon }=\ln A_{1}+n\ln\sigma -Q/RT(\alpha \sigma <0.8)\\ \ln\dot{\varepsilon }=\ln A_{2}+\beta \sigma -Q/RT(\alpha \sigma >0.8)\\ \ln\dot{\varepsilon }=\ln A+m\ln[\sinh(\alpha \sigma )]-Q/RT(\mathrm{All}\;\mathrm{stress})\\ \ln Z=\ln A+m\ln[\sinh(\alpha \sigma )] \end{array}\right.$$

where $\dot{\varepsilon }$ is the response speed, *Q* is the activation energy of exothermic reaction, *R* is the gas constant and A , n , β , α , m are constants, β=αn , respectively.

**Figure 9:**
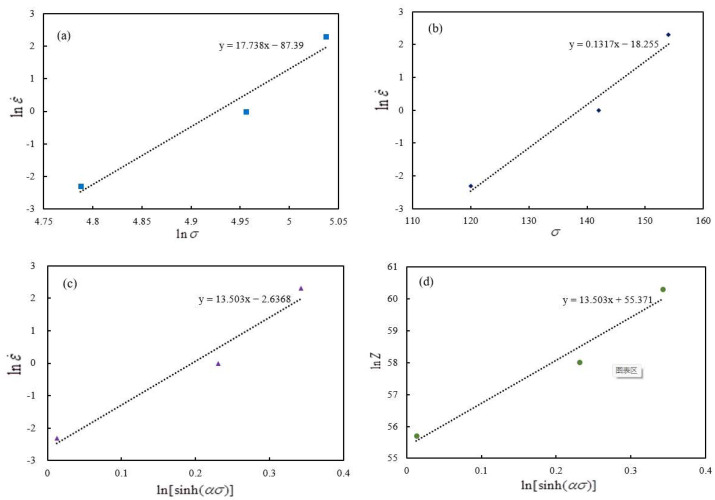

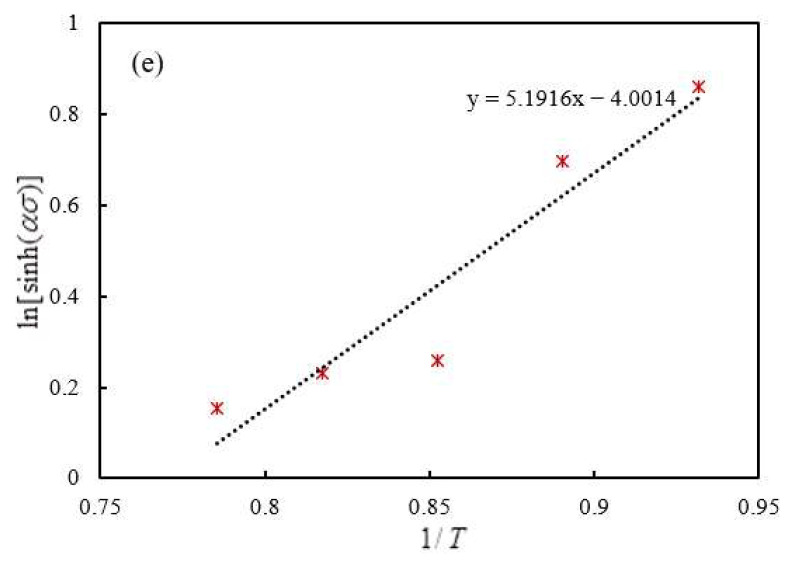
Sample 1# material constants determination chart: (**a**) *n*, (**b**) *β*, (**c**) *m*, (**d**) *A*, (**e**) *Q*.

Through high-temperature compression experiments, stress–strain relationships under different conditions were obtained. Based on the established high-temperature constitutive model, the determination of material constants involves fitting the deformation of stress–strain relationships under different conditions. Ultimately, the material constants are represented by the slope or intercept of the fitted lines. The fitting results are shown in Figure 9, where (a)~(e) correspond to parameters *n*, *β*, *m*, *A*, *Q*, respectively.

By substituting the computed material constants into the Arrhenius equation and rearranging, we can obtain the constitutive model for sample 1#, as shown in Equation (8).

(8) $$\left\{\begin{array}{l} \sigma =\frac{1}{\alpha }\mathrm{arc}\sinh\left(\sqrt[{m}]{\frac{\dot{\varepsilon }\exp\left(Q/RT\right)}{A}}\right)\\ \alpha =0.00742,\;m=13.503,\;Q=582830,\;A=1.115\times 10^{24} \end{array}\right.$$

To clarify the ultimate impact of the strengthening effect on the deformation, the obtained strengthening effect model can be combined. The control group can be set as not considering the strengthening effect and separately constructing its constitutive model. Finally, it can be incorporated into the deformation simulation model to compare the contribution of the strengthening effect to stress and its influence on the deformation as shown in Equation (9), where $\sigma^{\prime }$ is the stress of control group.

(9) $$\sigma^{\prime }=\sigma -\sigma_{Orowan}$$

**Figure 10:**
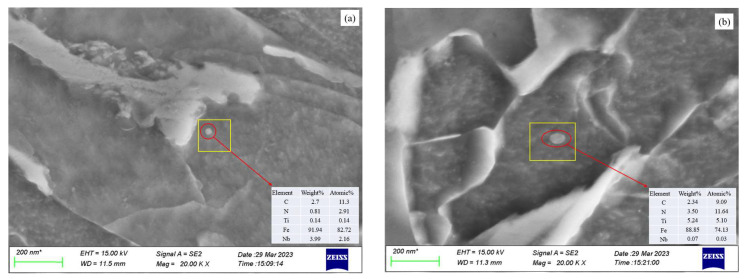
Microstructure of precipitates observed under SEM after stress relaxation at 900 °C (**a**) and 1000 °C (**b**) for sample #1 with the element, mass fraction (Weight) and atomic ratio (Atomic) of the precipitation.

Figure 10 shows the microstructure of the precipitates observed under SEM after stress relaxation of Sample #1 at 900 °C (a) and 1000 °C (b). It can be observed that the precipitates are composite precipitates of Nb and Ti, and the size of the precipitate particles increases with increasing rolling temperature. The volume fraction of the precipitated phases increases with decreasing temperature, which is consistent with the results obtained from the calculations and analysis of the theoretical model.

**Figure 11:**
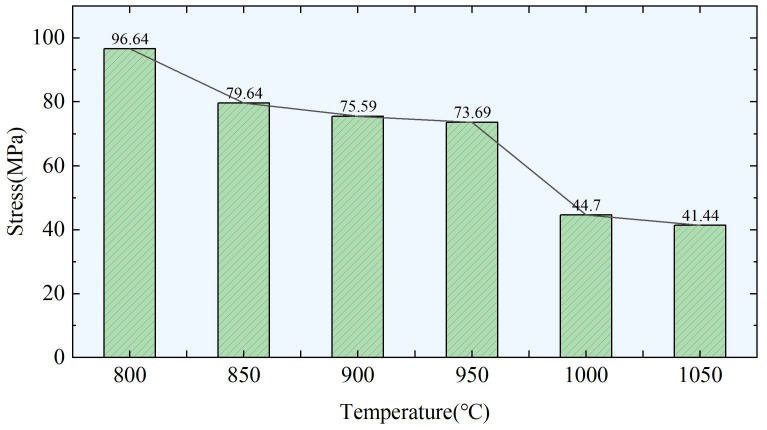
Strengthening effect of precipitation in sample #1 after precision rolling.

Considering that actual production is a continuous cooling process, the continuous process can be considered as a finite number of isothermal processes. The average diameter of the precipitated phases at each temperature ranges from 7.5 nm to 16.5 nm. Therefore, taking the average value of 10 nm and substituting it into the theoretical model of the strengthening effect of precipitation, the final strengthening effect is shown in Figure 11. The contribution of precipitated austenite in Sample #1 to the deformation resistance is approximately tens of megapascals, and as the temperature decreases, the strengthening effect increases. It reaches approximately 40 MPa above 1000 °C and can reach 96.6 MPa at 800 °C. By incorporating the establishment of a temperature field, the deformation resistance of microalloyed steel can be obtained at different temperatures and positions. This can be further applied to an FEM model to determine variations in the strip shape.

## 4. Temperature Field and FEM Model

### 4.1. Construction of the Rolling Temperature Field

In the hot strip rolling production process, an accurate calculation model of the strip temperature field is crucial for ensuring the quality of the strip in terms of shape, dimensional accuracy, and microstructure. Therefore, before conducting FEM rolling simulations, it is necessary to construct the rolling temperature field. Due to the relatively small influence of precipitation strengthening on temperature, it can be approximately neglected during the rolling process. Therefore, the finite difference method is adopted to solve the temperature field [31].

**Figure 12:**
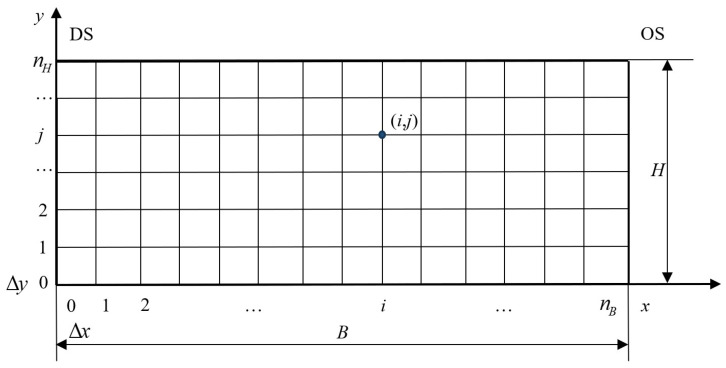
Methods for temperature field grid partitioning.

Since the length of the rolled material is much larger than its width and thickness, heat transfer in the longitudinal direction is relatively small. Furthermore, temperature control techniques in the longitudinal direction have become mature, and the temperature difference can be controlled within 10 °C. Therefore, when studying the temperature field of the strip, the heat transfer in the longitudinal direction can be neglected, and the three-dimensional problem can be simplified into a two-dimensional problem [32]. The first step is to discretize the spatial coordinates. The grid partitioning method is shown in Figure 12. *B* and *H* are the strip width and thickness, respectively. *B* × *H* cross-sectional area is divided into many rectangular grids with equal spacing Δ*x* and Δ*y* along the *x* and *y* directions, respectively. This generates *n_B_* and *n_H_* grids, where *i* is the node number in the *x* direction and *j* is the node number in the *y* direction.

Assuming an internal node (*i*, *j*) undergoes a temperature change from $T_{i,j}^{k}$ at time step *k* to $T_{i,j}^{k+1}$ at time step *k +* Δ*t*, the following Equations (10) and (11) can be used to calculate the temperature for the first Δ*t*/2 time step and the last Δ*t*/2 time step. A quick solution can be achieved using the tridiagonal matrix algorithm.

(10) $$\left(\begin{array}{ccccccc} b_{0} & c_{0} & & & & & \\ a_{1} & b_{1} & c_{1} & & & & \\ & \ddots & \ddots & \ddots & & & \\ & & a_{i} & b_{i} & c_{i} & & \\ & & & \ddots & \ddots & \ddots & \\ & & & & a_{n_{B}-1} & b_{n_{B}-1} & c_{n_{B}-1}\\ & & & & & a_{n_{B}} & b_{n_{B}} \end{array}\right)\left(\begin{array}{c} T_{0,j}^{k+\frac{1}{2}}\\ T_{1,j}^{k+\frac{1}{2}}\\ \vdots \\ T_{i,j}^{k+\frac{1}{2}}\\ \vdots \\ T_{n_{B}-1,j}^{k+\frac{1}{2}}\\ T_{n_{B},j}^{k+\frac{1}{2}} \end{array}\right)=\left(\begin{array}{c} d_{0}\\ d_{1}\\ \vdots \\ d_{i}\\ \vdots \\ d_{n_{B}-1}\\ d_{n_{B}} \end{array}\right)$$

(11) $$\left(\begin{array}{ccccccc} b_{0} & c_{0} & & & & & \\ a_{1} & b_{1} & c_{1} & & & & \\ & \ddots & \ddots & \ddots & & & \\ & & a_{j} & b_{j} & c_{j} & & \\ & & & \ddots & \ddots & \ddots & \\ & & & & a_{n_{H}-1} & b_{n_{H}-1} & c_{n_{H}-1}\\ & & & & & a_{n_{H}} & b_{n_{H}} \end{array}\right)\left(\begin{array}{c} T_{i,0}^{k+1}\\ T_{i,1}^{k+1}\\ \vdots \\ T_{i,j}^{k+1}\\ \vdots \\ T_{i,n_{H}-1}^{k+1}\\ T_{i,n_{H}}^{k+1} \end{array}\right)=\left(\begin{array}{c} d_{0}\\ d_{1}\\ \vdots \\ d_{j}\\ \vdots \\ d_{n_{H}-1}\\ d_{n_{H}} \end{array}\right)$$

where $T_{i,j}^{k+\frac{1}{2}}$ is the temperature of the control volume corresponding to node (*i*, *j*) after Δ*t*/2 time. The coefficients are as shown in Equation (12).

(12) $$\left\{\begin{array}{l} a=-f_{x}\\ b=2(g+f_{x})\\ c=-f_{x}\\ d=f_{y}T_{i,j-1}^{k}+2(g-f_{y})T_{i,j}^{k}+f_{y}T_{i,j+1}^{k}+q_{in}\\ f_{x}=\frac{\lambda }{\Delta x^{2}}\\ f_{y}=\frac{\lambda }{\Delta y^{2}}\\ g=\frac{\rho c}{\Delta t} \end{array}\right.$$

where *c* is the specific heat capacity, *ρ* is the density, *λ* is the thermal conductivity, *q_in_* is the heat flux density from internal heat sources, *T* is the temperature value, *x* is the coordinate in the width direction, and *y* is the coordinate in the thickness direction, respectively. Then, the temperature value of any node at any given time in the steel strip can be obtained by solving the system of equations, which in turn allows for the determination of the temperature distribution throughout the entire steel strip.

**Figure 13:**
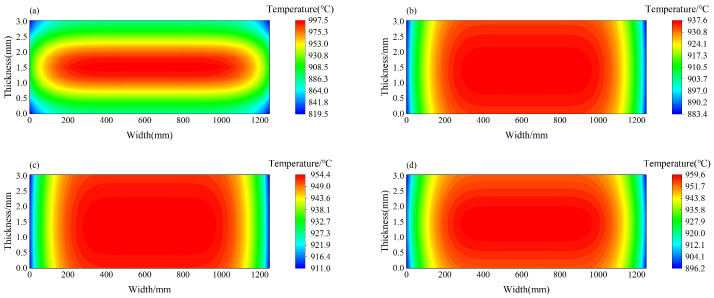
When the activation indicators for cooling water for F2, F3, F4, and F5 are (0111) (**a**), (1011) (**b**), (1101) (**c**), and (1110) (**d**), the temperature distribution at the exit section of F6 frame.

Utilizing the theory model and computational process, a temperature field calculation model for strip steel in the finishing frame zone is established using the C++ programming language. The practical process equipment parameters of a certain hot rolling production line are taken as an example to calculate the distribution of exit section temperature in four different rolling states on the F6 stand. The simulated process parameters are shown in Table 2. The calculation results are shown in Figure 13, where the four operating conditions correspond to the opening indicators of cooling water for F2, F3, F4, and F5 as (0111) (a), (1011) (b), (1101) (c), and (1110) (d). In the indicators, 1 represents open and 0 represents close.

In all four different operating conditions, the maximum temperature is approximately 950 °C, and the temperature drop at the edges exceeds 50 °C. However, the temperature difference in the thickness direction is not significant, so its influence on precipitation can be neglected. To simulate the rolling conditions more realistically, subsequent experimental simulations and simulations are controlled within this temperature range.

### 4.2. Construction of Elastic-Plastic FEM Model

This paper takes a 1250 mm hot tandem rolling four-roll precision mill as an example. The simulation analysis and calculation model are established using the elastic-plastic FEM with ABAQUS 2022 software. The working rolls and support rolls are considered elastic materials, while the slab material is considered an elastic-plastic material.

**Figure 14:**
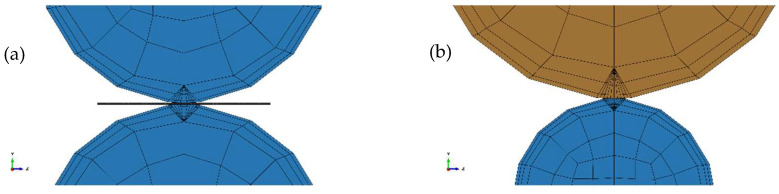
The mesh between the working roll and the strip (**a**), and between the working roll and the support roll (**b**).

The selection of mesh element types adopts the 8-node hexahedral linear reduced integration element C3D8R. The working rolls and support rolls are partitioned using the method of locally dividing the artificial sketch. The size of the boundary elements is the same as that of the slab, while the internal mesh elements are larger. The contact surface between the working roll and support roll, as well as the boundary mesh of the steel strip, are locally refined to improve the computational accuracy. In Figure 14a,b show the mesh between the working roll and the strip, and between the working roll and the support roll. Furthermore, a set of nodes on the upper surface of the cross-section in the middle of the contour node collection of the slab is selected and created in the components for output analysis. The key parameters for the rolls and steel strip are shown in Table 3.

The model consists of five analysis steps, with the initial step being static explicit, while the remaining steps are static implicit. These steps include small reduction, large reduction, application of bending force, application of tension, and rolling. The duration of each analysis step is set to 1. The initial increment step is set to 0.01, with the minimum increment step at 1 × 10^−5^, maximum increment step at 1, and a maximum number of increment steps at 10,000. During operation, the working rolls are only allowed to be pressed and rotated in the thickness direction by the supporting rolls. Therefore, in the first four analysis steps, the degrees of freedom (U1, U3, UR1, UR2, UR3) of the working rolls are constrained. The restriction on UR1 is lifted in the rolling analysis step. The supporting rolls are allowed to be pressed down only in the thickness direction without considering rotation. Hence, the degrees of freedom (U1, U3, UR1, UR2, UR3) of the supporting rolls are restricted. Meanwhile, in the small reduction analysis step, the displacement of U2 is set to 0.1, and in the large reduction analysis step, it is set to 0.5. In the analysis step of applying bending force, it is set to 1.2 and maintained at that value. In the case of the strip steel, before it moves along the *Z*-axis with the rotation of the working rolls, the front end face of the strip steel is fixed to prevent tilting during roll pressure. Therefore, in the first four analysis steps, the degree of freedom (U3) of the front end face of the strip steel is restricted, and this restriction is lifted in the rolling analysis step.

Under non-uniform lateral temperature distribution, variations in precipitation states lead to differences in the mechanical properties of materials. Specifically, the heterogeneity in the transverse microstructure distribution gives rise to disparities in mechanical performance, resulting in different deformation resistance under various conditions. To customize the material’s hardening behavior during plastic deformation, the SDVINI and UHARD subroutines are employed utilizing the Fortran programming language.

### 4.3. Design of FEM Model Work Conditions

To consider the effect of precipitation on the mechanical properties of the strip steel at different temperatures, as well as the transverse temperature difference that exists in actual production, the impact of uneven distribution of mechanical properties caused by precipitation at different temperatures on the strip steel is analysed. According to the temperature field calculation results, the strip steel is set to have a central temperature of 1000 °C and 950 °C, with transverse temperature differences of 0 °C, 40 °C, 80 °C, and 120 °C. The temperature distribution is characterized by an eighth-degree polynomial function, and with the help of analytical functions, the transverse temperature field distribution is input into the FEM model as shown in Equation (13).

(13) $$T(x)=T(0)-\Delta T{\left(x/d_{0}\right)}^{8}$$

where *x* is the transverse coordinate of the strip steel in millimeters, *T*(0) is the central temperature, which can be either 1000 °C or 950 °C, Δ*T* is the temperature drop, which can be 0 °C, 40 °C, 80 °C, or 120 °C, and *d*_0_ is the width of the strip steel, which can be either 1000 mm or 1250 mm, respectively, for simulating the rolling process of F6.

To compare and analyse the influence of uneven mechanical properties caused by uneven distribution of precipitate strengthening on the cross-sectional profile of the strip steel, five different working conditions were mainly designed for comparison. The impact of uneven transverse distribution of mechanical properties caused by precipitation on the strip steel is considered, as shown in Table 4.

## 5. Results and Discussion

Crown refers to the difference between the thickness at the midpoint of a strip and the average thickness at two marked points on the side edges, serving as an important parameter to characterize the strip shape. In this paper, the measure of crown is used to assess the strip shape. As shown in Equation (14), where *C* is the crown, is the thickness at the midpoint of the strip width, and are the thickness 40 mm away from the strip edge on the operating side and the transmission side, respectively.

(14) $$C=h_{c}-\frac{h_{w}+h_{d}}{2}$$

**Figure 15:**
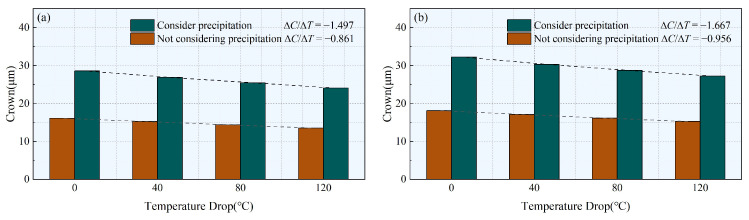
Influence of considering and not considering precipitation on crown. (**a**) Centre temperature is 1000 °C (Corresponding condition 1 and condition 4); (**b**) Centre temperature is 950 °C (Corresponding condition 2 and condition 5).

Figure 15 shows the simulated crown of a 1500 mm wide strip under different operating conditions. Figure 15a presents a comparison of the crown considering precipitation and without precipitation for operating conditions 1 and 4, with a center temperature of 1000 °C. Figure 15b displays a comparison of the crown considering precipitation and without precipitation for operating conditions 2 and 5, with a center temperature of 950 °C. In Figure 15a, when the center temperature is 1000 °C and the temperature drop is 0 °C, the stress variation of 44.7 MPa results in a crown increase of 12.46 µm. However, comparing Figure 15a with Figure 15b, with the same temperature drop of 0 °C and a temperature difference of 50 °C, only a slight increase in crown of 2.5 µm is observed. The effect of precipitation strengthening on crown is far greater than the influence of temperature reduction. In Figure 15a, when the center temperature is 1000 °C and the temperature drop is 0 °C, the difference in crown between considering precipitation and without precipitation is 12.46 µm, while at 120 °C it is 10.53 µm, reducing by 1.93 µm. In Figure 15b, when the center temperature is 950 °C and the temperature drop is 0 °C, the difference is 14.1 µm, while at 120 °C it is 11.96 µm, reducing by 2.14 µm. This indicates that the lower the overall temperature of the strip, the greater the influence of precipitation strengthening on the uneven distribution of thickness in the strip, and due to the lower temperature at the rear racks, it is more prone to shape issues under the influence of precipitation strengthening.

The sensitivity of crown to temperature differences can be represented by ∆*C*/∆*T* (The ratio between variations in crown and variations in temperature). The absolute value of ∆*C*/∆*T* is greater when considering precipitation than when not considering precipitation, indicating that the crown is more sensitive to temperature differences when precipitation is considered. As the overall temperature decreases, the absolute value of ∆*C*/∆*T* increases. However, at a centre temperature of 950 °C, compared to 1000 °C, the absolute value of ∆*C*/∆*T* increases by 0.17 when considering precipitation, while it only increases by 0.095 without considering precipitation. This indicates that considering precipitation makes the crown more sensitive to overall temperature changes.

**Figure 16:**
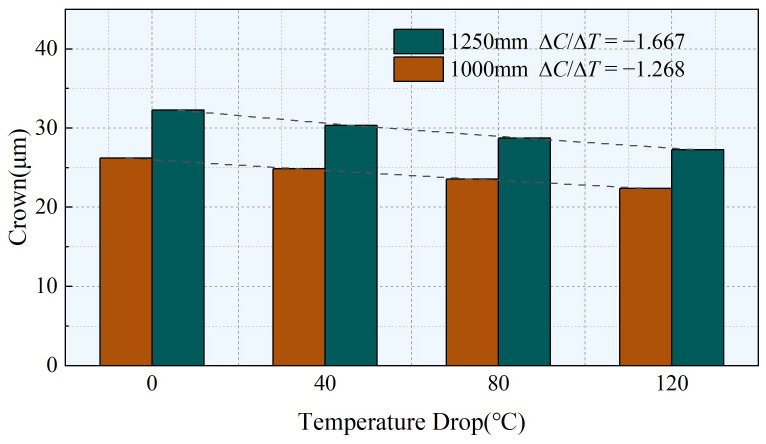
Influence of strip steel width on crown under different temperature differences.

As shown in Figure 16, the simulation results illustrate the crown under different widths for working conditions 2 and 3. The narrowing of the width of the strip leads to the application of the same tension on a smaller cross-sectional area. Consequently, the range of the internal tension is reduced, resulting in a weakened effect of the internal tension. Simultaneously, this also causes an imbalance in the lateral forces, thereby reducing the bending stiffness of the strip and overall decreasing its crown. Under the influence of reinforcement, the resistance to deformation in the marginal area increases, providing a stronger resistance against deformation. Similarly, the changes in crown caused by the same temperature drop are relatively small. When the width is 1250 mm, the crown change caused by temperature drops of 0 °C and 120 °C is 5.01 µm, whereas it is 3.80 µm when the width is 1000 mm. Furthermore, the absolute value of ∆*C*/∆*T* increases by 31.47% when the width is 1250 mm compared to when it is 1000 mm, indicating a decreased sensitivity of crown to temperature drops as the width decreases.

## 6. Conclusions

This paper predicts the influence of precipitate behavior during precision rolling of Nb-Ti microalloyed steel on strip shape through calculations of the theoretical model and FEM. The following conclusions can be drawn:

1. From the high-temperature compression experiment, it is known that due to the large difference in Nb and Ti element content, sample 1# has a maximum true stress about 20% greater than sample 2#, and it exhibits greater deformation resistance during hot rolling.
2. After calculating the theoretical model of precipitation, it was known that the content of microalloying elements significantly affects the precipitation rate and the volume fraction of precipitates. The enhancement effect of precipitation on the strength in Sample 1# varies significantly, reaching a maximum of 96.6 MPa. By comparing with the control group, it can be concluded that precipitates are an important influencing factor for the shape of hot rolling strips in steel strips.
3. The strip temperature decreases with the increase of the mill stand, and precipitation strengthening will exacerbate the uneven distribution of transverse strength of the strip, causing uneven thickness distribution and easy occurrence of strip shape problems. In addition, considering the sensitivity of crown to the overall temperature during precipitation, the sensitivity of crown to temperature drop decreases as the strip width decreases.

## Figures and Tables

**Table 1:** Chemical composition (wt%) of the experimental steel.

Steel Sample	C	Si	Mn	Ti	S	Nb	P	N	Fe
1#	0.0712	0.353	1.193	0.0026	0.0019	0.0513	0.011	0.0027	Bal.
2#	0.0687	0.015	0.857	0.0008	0.0046	0.0136	0.009	0.0036	Bal.

**Table 2:** The process parameters for simulating the temperature field model of strip steel.

Parameter	F1	F2	F3	F4	F5	F6
Entry thickness (mm)	42.95	17.81	11.33	7.62	4.37	3.25
Exit thickness (mm)	17.81	11.33	7.62	4.37	3.25	3.15
Exit velocity (m/s)	0.19	1.16	2.19	3.55	5.58	7.63
Rolling force (kN)	2034	2359	2555	2738	2092	1936
Work roll diameter (mm)	820	806	750	758	582	554
Roll center distance (mm)	5.5					
Length of cooling water before and after rolling (mm)	0.5					
Length of cooling water between frames (mm)	1					
Width of cooling water between frames (mm)	1500					
Width(mm)	1250					

**Table 3:** Key parameters of rolls and steel strip.

Strip			Working Roll		Supporting Roll	
Entry thickness	Exit thickness	Width	Diameter	Length	Diameter	Length
5 mm	3 mm	1250/1000 mm	850 mm	2550 mm	1600 mm	2250 mm

**Table 4:** Work condition design.

Condition Code	Central Temperature (°C)	Strip Width (mm)	Temperature Drop (°C)
1-Consider precipitation	1000	1250	0/40/80/120
2-Consider precipitation	950	1250	0/40/80/120
3-Consider precipitation	950	1000	0/40/80/120
4-Not Consider Precipitation	1000	1250	0/40/80/120
5-Not Consider Precipitation	950	1250	0/40/80/120

## Data Availability

The datasets generated during and/or analyzed during the current study are not publicly available due to the sensitive nature of the industry collaboration, but are available from the corresponding author on reasonable request.
